# Treating pyruvate kinase deficiency with Mitapivat: a short communication

**DOI:** 10.1097/MS9.0000000000000759

**Published:** 2023-05-03

**Authors:** Muhammad Omar Larik, Moeez Ibrahim Shiraz, Muhammad Ashhal Iftekhar, Seemin Afshan Shiraz, Maira Shiraz

**Affiliations:** a Dow International Medical College, Dow University of Health Sciences, Karachi, Pakistan; b Mediclinic Parkview Hospital, Dubai; c Pamir Private School, Sharjah, United Arab Emirates

Pyruvate kinase deficiency (PKD) is an inherited haemolytic disease characterized by a red blood cell (RBC) enzyme defect and is the second most common cause of chronic haemolytic anaemia^1,2^. It is considered to be a rare congenital blood disorder, with the prevalence of clinically diagnosed patients reaching between 3.2 and 8.5 million in the Western regions. However, it is expected for the total number of PKD sufferers to peak as high as 51 per million, illustrating that the majority of patients remain undiagnosed throughout their lives^3^. The clinical manifestations displayed by PKD can be varied. Patients may range from being completely asymptomatic due to compensation to an exceedingly severe manifestation characterized by transfusion-dependent anaemia^4^. Diagnosis typically occurs during infantile or childhood years, although diagnosis in adulthood is possible in patients with compensated haemolysis. Common complications of PKD include extramedullary hematopoiesis, iron overload, pulmonary hypertension, gallbladder-related pathologies, and more^5^.

Current management guidelines consist of supportive therapy, iron chelation, and splenectomy. Traditionally, red cell transfusions, paired with iron chelators, are preferred during infancy and childhood in cases of significant symptomatic anaemia in which the quality of life is remarkably impacted^4^. Splenectomy, an invasive surgical procedure, can also be conducted in patients who continue to suffer from symptomatic anaemia despite highly frequent transfusions^4^. This procedure is not recommended for infancy or the elderly, but remains an appropriate opportunity for children and adults. However, with the current ongoing active research in the field of pyruvate kinase activators, a wide array of prospects have been unlocked in patients suffering from PKD.

The enzyme pyruvate kinase is responsible for the conversion of phosphoenolpyruvate to pyruvate, resulting in the generation of adenosine triphosphate (ATP). While most cells generate ATP via aerobic pathways, one of the exceptions is the RBC, which lacks mitochondria necessary for the aerobic glycolytic pathway^6^. Thus, RBCs are completely reliant on the anaerobic glycolytic pathway for energy generation, in order to maintain their structural integrity^6^. The defective process in PKD involves the enzyme pyruvate kinase, which fails to catalyze the conversion of phosphoenolpyruvate to pyruvate. Subsequently, there is a decreased production of ATP, resulting in RBC degradation and the landmark clinical sign of congenital haemolytic anaemia^6^.

Mitapivat (AG-348) is a novel first-in-class allosteric activator of pyruvate kinase. The mechanism of action involves pyruvate kinase enzymes being activated allosterically by the binding of Mitapivat in lieu of fructose bisphosphate^6^. Mitapivat received Food and Drug Administration (FDA) approval in February 2022, for oral administration in adults suffering from PKD, thalassaemia, and sickle cell disease^6,7^. In a randomized controlled trial (RCT) conducted on patients with PKD, the efficacy of Mitapivat was compared with the placebo^8^. The results of this successful trial demonstrated positive outcomes, including a mean increase of haemoglobin by 1.8 g/dl, a mean decrease of reticulocyte count of −10.1%, and a mean decrease of lactate dehydrogenase by −70.8 U/l^8^. Furthermore, both treatment arms had the same number of total adverse events, although patients receiving Mitapivat were more prone to encountering treatment-related adverse events^8^.

The positive results reveal the efficacious nature of Mitapivat, highlighting this novel drug as a suitable treatment option for patients suffering from PKD. These findings are further consolidated by other similar clinical trials conducted utilizing Mitapivat^9,10^. The current treatment strategy largely revolves around the use of frequent and routine RBC transfusion, which carries the risk of complications including the transmission of infectious agents, immune system suppression, circulatory overload, and errors in administration^11^. Furthermore, the invasive splenectomy procedure may result in a myriad of complications, ranging from post-splenectomy sepsis to post-splenectomy thromboprophylaxis, in addition to the typical post-surgical complications experienced by patients^12^. Additionally, exploration of other novel treatment strategies has led to the discovery of the use of stem cell transplantation (bone marrow transplantation), with the expectancy of increasing stable RBC production in PKD patients^13^. However, with current limited research alongside the increased mortality detected in certain studies, the use of stem cell transplantation has not been generally indicated in PKD patients, especially in certain age groups^13^. Therefore, with the present availability of research assessing the numerous treatment strategies of PKD, Mitapivat serves to be an excellent opportunity for treatment of patients suffering from PKD. This can be exemplified by the favourable safety profile achieved in recent RCTs^8–10^, providing a safer treatment choice for PKD sufferers, especially in comparison with other treatment options available as per the current clinical guidelines.

Further extensive studies are required to determine whether Mitapivat can solely replace the current treatment guidelines for PKD, on account of extended clinical trials assessing the long-term efficacy and safety. Moreover, the role of Mitapivat in the treatment of paediatric cases of PKD should be assessed via comprehensive RCTs, including the determination of efficacy, safety, and dose-response relationships based on different age groups.

## Ethical approval

Not applicable.

## Consent

Not applicable.

## Sources of funding

None.

## Conflicts of interest disclosure

None.

## Provenance and peer review

Not commissioned, externally peer-reviewed.
